# Assessment of Sexual Violence Risk Perception in Men Who Have Sex With Men: Proposal for the Development and Validation of “G-Date”

**DOI:** 10.2196/57600

**Published:** 2024-08-19

**Authors:** D J Angelone, Damon Mitchell, Brooke Wells, Megan Korovich, Alexandra Nicoletti, Dustin Fife

**Affiliations:** 1 Department of Psychology Rowan University Glassboro, NJ United States; 2 Department of Criminology & Criminal Justice Central Connecticut State University New Britain, CT United States; 3 Center for Human Sexuality Studies Widener University Chester, PA United States

**Keywords:** sexual violence, risk perception, laboratory analog, dating and sexual networking apps, men who have sex with men

## Abstract

**Background:**

Sexual violence (SV) is a significant problem for sexual minorities, including men who have sex with men (MSM). The limited research suggests SV is associated with a host of syndemic conditions. These factors tend to cluster and interact to worsen one another. Unfortunately, while much work has been conducted to examine these factors in heterosexual women, there is a lack of research examining MSM, especially their SV risk perception. Further, MSM are active users of dating and sexual networking (DSN) mobile apps, and this technology has demonstrated usefulness for creating safe spaces for MSM to meet and engage partners. However, mounting data demonstrate that DSN app use is associated with an increased risk for SV, especially given the higher likelihood of using alcohol and other drugs before sex. By contrast, some researchers have demonstrated that DSN technology can be harnessed as a prevention tool for HIV; unfortunately, no such work has progressed regarding SV.

**Objective:**

This study aims to (1) use qualitative and quantitative methods to tailor an existing laboratory paradigm of SV risk perception in women for MSM using a DSN mobile app framework and (2) subject this novel paradigm to a rigorous validation study to confirm its usefulness in predicting SV, with the potential for use in future prevention endeavors.

**Methods:**

To tailor the paradigm for MSM, a team of computer scientists created an initial DSN app (G-Date) and incorporated ongoing feedback about the usability, feasibility, and realism of this tool from a representative sample of MSM. We used focus groups and interviews to assist in the development of G-Date, including by identifying relevant stimuli, developing the cover story, and establishing the appropriate study language. To confirm the paradigm’s usefulness, we are conducting an experimental study with web-based and face-to-face participants to determine the content, concurrent, and predictive validities of G-Date. We will evaluate whether certain correlates of SV informed by syndemics and minority stress theories (eg, history of SV and alcohol and drug use) affect the ability of MSM to detect SV risk within G-Date and how paradigm engagement influences behavior in actual DSN app use contexts.

**Results:**

This study received funding from the National Institute on Alcohol Abuse and Alcoholism on September 10, 2020, and ethics approval on October 19, 2020, and we began app development for aim 1 immediately thereafter. We began data collection for the aim 2 validation study in December 2022. Initial results from the validation study are expected to be available after December 2025.

**Conclusions:**

We hope that G-Date will enhance our understanding of factors associated with SV risk and serve as a useful step in creating prevention programs for this susceptible population.

## Introduction

### Background

Sexual violence (SV) includes any nonconsensual oral, anal, or vaginal contact or penetration in which the perpetrator uses force, intimidation, coercion, or other means (eg, purposeful intoxication) to impose sexual contact [1]. SV is an umbrella term that encompasses a variety of experiences (eg, sexual assault and rape) between strangers, relatives, acquaintances, or intimate partners [2]. National surveys indicate lifetime prevalence rates of approximately 20% for penetrative SV and 44% for nonpenetrative SV in women and 2% for penetrative SV and 23% for nonpenetrative SV in men [3-5]. While much of the literature focuses on heterosexual individuals, SV is more prevalent among sexual minorities, including men who have sex with men (MSM) [6-8]. Estimates of lifetime exposure to SV vary considerably according to definitions and samples, with between 26% and 67% of MSM reporting such experiences [9]. Further, intimate partner violence (IPV), which often includes or is associated with SV [10,11], is also highly prevalent among MSM, with rates of IPV exposure ≥33% [12]. Finally, alcohol use by the SV survivor, perpetrator, or both is a common risk factor noted across studies [13].

The prevalence of exposure to SV among MSM is not surprising, given that sexual minority men experience a clustering of challenging conditions. Syndemics theory posits that negative social conditions cluster and interact to worsen one another [14,15]. In fact, SV is associated with a range of other syndemic conditions, including substance use, mental health disorders, IPV, sexual compulsivity, sexual risk behavior, and HIV transmission [7,16-22]. Likely a common basis for many psychosocial syndemic conditions in MSM [23], minority stress theory posits that the expectations for, experiences of, and internalization of discrimination and prejudice contribute to health disparities and outcomes [23-26], including SV [9,27]. In fact, minority stress is consistently associated with a variety of syndemic conditions, such as alcohol and other drug use [28,29] and SV or IPV [13,18]. Taken together, these theories suggest that a variety of constructs might be associated with SV risk; therefore, these factors will be used as mechanisms to validate the proposed laboratory paradigm.

### Dating and Sexual Networking Mobile App Use Among MSM

In addition to heightened susceptibility to SV and its clustering with other syndemic conditions, MSM also frequently participate in dating and sexual networking (DSN) via mobile apps and other web-based venues [30,31]. Research indicates high rates of sexual risk behavior, more partners among internet and DSN app users [32,33], and a higher likelihood of using alcohol or other drugs before sex [33,34], thus highlighting the potential for exposure to SV with men met via DSN apps. While the prevalence of SV by partners met via DSN apps is unknown, research, media, and law enforcement reports suggest it may be common [35]. One study found that 9.3% of women and 11.4% of men reported SV with someone they met on a dating site or mobile app [36]. Research across gender and sexual orientation groups indicates a higher risk of past-year SV among those who use dating apps than among those who do not, although studies have not examined exposure to SV by partners specifically met via dating apps [37,38]. Similarly, a survey of DSN app–using MSM found that 11% reported lifetime experience of sexual IPV [39], although not necessarily perpetrated by DSN app–using men. Finally, a recent study of violence related to DSN use in MSM found that 33% reported some exposure to physical violence or SV by someone they met on a DSN app. Moreover, nearly 48% noted other MSM they know who have experienced physical violence or SV by someone they met on a DSN app [40].

Despite limited prevalence data, DSN app–met partners pose a risk for exposure to SV for several reasons: the anonymity inherent in web-based spaces, meeting in private locations, a lack of disclosure of whereabouts to others for privacy reasons or stigma avoidance, the potential for perpetrators to target and access potential targets, disinhibition regarding sharing personal details in web-based spaces, and heightened expectations for sex [37]. It is also common for MSM to use apps while under the influence of alcohol or drugs, and many individuals use substances when they meet in person [6,37]. Further, MSM are concerned for their physical or sexual safety when seeking and meeting partners in web-based venues [41]. DSN app use represents a unique opportunity for MSM to detect risk factors for SV, screen potential partners, and control their interactions [42]. MSM engage in sexual preference and risk negotiations in web-based spaces [43,44]. When MSM use alcohol or drugs while vetting potential partners, their ability to detect risk may also be impaired. Because MSM use DSN apps to make sexual decisions and negotiate risk, additional research is needed to understand the information that guides their decisions about sexual activity with partners met through DSN apps.

### Identifying Risk Factors for SV Perpetration Among MSM

While little is known about SV risk perception and negotiation in DSN environments, the literature has identified specific risk factors among men. Specifically, for this project, we focused on problematic alcohol and drug use, antisociality, and the internalization of homophobia. First, alcohol and drugs are common in dating and sexual encounters with DSN app–met partners [45]. Among MSM, alcohol and drug use in general, and in sexual situations specifically, predicts SV perpetration [46-49]. As such, alcohol and drug use cues may help identify potential partners more likely to perpetrate SV.

Second, among heterosexual men, signs of antisociality, such as controlling behaviors [50], low levels of agreeableness [51], and hypermasculinity (and perhaps also a preference for these characteristics in potential partners), predict SV perpetration [52]. Among heterosexual men, masculine discrepancy stress, where men perceive they do not meet traditional standards of masculinity, is associated with IPV perpetration [53], sexual risk behavior, and alcohol and drug use [54], all of which are risk factors for SV perpetration. Third, and unique to MSM, minority stressors, such as internalized homophobia, or anticipating sexual orientation prejudice or discrimination correlates with perpetration [55]. That said, it is important to note that while such factors may increase risk for exposure to SV or affect one’s decision-making and risk perception, it is never a person’s fault for experiencing SV.

### Existing Measures of SV Risk Perception

In general, researchers examining SV risk perception have studied heterosexual dyads and relied on vignettes. For example, the potential perpetrator in a vignette may use alcohol or attempt to isolate a potential target, and participants indicate the point when they would begin to feel uncomfortable and when they report wanting to leave the situation [56,57]. Failing to report discomfort during the reading of a vignette and delayed exit are conceptualized as poor SV risk perception. For example, participants can be presented with a description of a first date and asked to rate the level of risk associated with several cues described in the date, such as a man paying for the date, alcohol intoxication, and making sexual comments [58,59]. This methodology has been expanded to include audio or video vignettes depicting an interpersonal situation in which participants are asked to project themselves and predict how they would respond [60,61]. Researchers then determine whether participants identify the threats and remove themselves from the hypothetical situation. The longer participants take to indicate that the man should stop his sexual advances, the higher the response latency and the poorer the SV risk perception.

Although SV risk cues can range from being ambiguous to blatant, most vignette scenarios portray clear cues and focus on high-risk situations. Further, many risk perception paradigms adhere to a third-person perspective; therefore, the risk may not be immediate to the participant, and their appraisals remain hypothetical. These measures may lack ecological validity: assumptions of how one *might* act may not accurately reflect one’s *actual* behaviors when confronted with the same situation in reality. Therefore, stronger tests of risk perception entail the examination of decision-making in an actual interpersonal interaction [59]. For example, in a paradigm, female participants engaged in a social interaction with a male confederate in a bar laboratory [62]. The researchers examined the impact of sexually ambiguous cues on participants’ nonverbal behaviors and perceptions of the confederate.

Given the limitations of existing risk perception paradigms and the paucity of measures that use a web-based medium, members of our team developed a paradigm (ie, EduDate) to examine SV risk perception in college women [63]. This novel web-based speed-dating paradigm assesses threat appraisals and behavioral reactions to SV risk. It provides an opportunity to study dispositional and environmental factors that influence the perception of clear and ambiguous risk cues. Naturally, the use of laboratory analog methods is limited by the nature of perpetration and by the need to engage in ethical and responsible research methods [64]. While the ultimate goal is to reduce SV, researchers are appropriately limited in their ability to mimic such behavior and examine the decision-making process of a potential target. As a laboratory method, there is a high degree of experimental control for EduDate, which provides strong internal validity. EduDate also approximates a real-world experience, which strengthens ecological validity. EduDate incorporates behaviors consistent with the literature about perpetrators and mimics situations where women respond to potential SV in real time.

EduDate was modeled in appearance and functionality after web-based dating services accessible via a computer rather than through a mobile app. Participating women are told the study involves “beta testing online speed-dating software.” Participants initially view a profile of a bogus dating candidate with whom they will be having a speed date and then rate the candidate across characteristics typical of speed dating (ie, physical attractiveness, social status, and likelihood to date). Next, each participant “interacts” with the candidate through a rigged chat-based speed date involving predetermined questions and responses. Once participants provide an answer to a question, they view the bogus dating candidate’s response, which presumably had been typed at the same time. The candidate’s responses become increasingly indicative of potential SV perpetration by the inclusion of risk cues empirically established for male-on-female SV. Risk perception is measured by the number of risk cues a participant tolerates, which represents behavioral response, and changes in the participant ratings from before to after the interaction with the dating candidate, which measures the threat appraisal [63]. There is evidence for the usefulness of EduDate to study SV risk perception, which supports adapting the paradigm to investigate SV risk perception in DSN app environments among MSM. However, potential differences in the risk factors for MSM compared to those for heterosexual women necessitate an empirically driven approach to ensure the stimuli used in the paradigm are tailored for MSM. In addition, the surge of technology, including the use of mobile apps, necessitates an infrastructure update to this laboratory-based tool.

### This Study

A total of 2 primary aims guide this study. Our first aim was to develop a rigged DSN app (ie, G-Date) that allows for the investigation of SV risk perception in MSM. Our work on the first aim included three objectives: (1) the production of an app whose appearance and functionality mimic those of contemporary real-time DSN apps used by MSM while actually being a predetermined and rigged system in which we could embed SV cues and assess participant engagement with these cues; (2) the development of stimulus materials to embed into the app; and (3) the refinement of the stimulus materials and app through an iterative process to ensure its appearance, functionality, and data collection capabilities were adequate for the purposes of the study. The EduDate paradigm (previously validated with heterosexual women) served as a foundation for designing this new paradigm.

Our second aim (which is in progress) is to validate the G-Date paradigm as a measure of SV risk perception in MSM. We will evaluate G-Date’s content validity by having participants undergo 2 speed dates on the app: one with a dating partner who displays SV risk cues and another with a dating partner who does not display SV risk cues. Next, we will compare participant engagement with, and ratings of, the risky versus the safe dating partners. We hypothesize that participants will end the speed date with the risky dating partner sooner than that with the safe partner and will rate the risky partner more negatively after the date. Per syndemics theory and the processes outlined in minority stress theory, we will evaluate G-Date’s concurrent validity by examining the extent to which participants’ existing risk factors for exposure to SV (eg, SV history and drug or alcohol use) predict their engagement with the risky versus the safe dating partners. We hypothesize that the length of participants’ speed date with the risky partner and their ratings of the risky candidate after the date will be predicted by syndemic and minority stress factors associated with SV. Finally, we will evaluate the predictive validity of G-Date by examining the extent to which participants’ engagement with the risky and safe dating partners predicts SV rates at 9-month follow-up. We hypothesize that the length of participants’ speed date with the risky partner and their ratings of the risky partner after the date will predict subsequent SV.

## Methods

### Ethical Considerations

This study received initial ethics approval from the Rowan University Institutional Review Board on October 19, 2020 (PRO-2020-25) and has continuously been approved since that time. All participants provide informed consent before engaging in the study and are able to opt out of the study for any reason. All data are deidentified using a 4-character code to match responses to the questionaries with the data stored in the app. All participants were paid US $40 for engaging in focus groups and interviews, US$30 for participation in part 1 of the validation study, and US $20 for part 2 of the validation study at 9-month follow up.

### Research Design Overview

#### Aims 1 and 2

The 2 aims of this research involve several points of qualitative and quantitative data collection. As presented in Figure S1 of Multimedia Appendix 1 [65-86], qualitative and quantitative designs were used to develop and refine the cover story; app procedures and functionality; and app materials, including photos and the question and answer (Q&A) scripts. We then used this information to finalize the app and research materials for use in aim 2.

The first aim of this project entailed the production of the G-Date app and protocol, creation of the stimulus materials to embed into the app, and subsequent refinement of both the stimulus materials and app to ensure their adequacy for the study. The production of the G-Date app and protocol involved working with an app development team led by a computer scientist and staffed by computer science graduate students. The team worked to make G-Date appear to be a contemporary DSN app geared for speed dating for MSM. We also wanted G-Date to be developed in such a way that other researchers could easily modify components of the app’s content and functionality for follow-up work in this area. G-Date uses a software platform similar to popular dating apps; therefore, its functionality and appearance would be familiar to those with even casual prior exposure to dating apps (Figure S2 in the Multimedia Appendix 1). The app was built to be compatible with iOS (Apple, Inc) devices (eg, iPad, iPhone, and MacBook). The protocol as experienced by the user involves four components: (1) profile setup, (2) the review of potential partners, (3) Q&A speed date, and (4) partner comparison (Textbox 1).

The creation of the stimulus materials involved the development of the (1) script for the Q&A speed date and (2) dating partner photos.

**Protocol components as experienced by users.**

**Profile setup**

Access to the app requires a username and password provided by the research team. Once logged into the app, participants set up their own profile, which involves (1) taking and uploading a headshot with the G-Date logo in the background; (2) providing information about themselves, such as that commonly found on dating apps for men who have sex with men (MSM), including age, relationship status, position (eg, top, bottom, or versatile), and community (eg, bear or jock); (3) providing information about the kind of person they are seeking (eg, age range, relationship status, position, and community); and (4) answering questions about their personality.

**Review of potential partners**

After the completion of their profile, participants view photos of 60 prospective dating partners who ostensibly meet their desired characteristics. The photos are headshots of men in the participant’s preferred age range, who vary in ethnic background, and who were rated as attractive in the pilot work (refer to the Dating Partner Photos section for more information on photos). Embedded into each photo is a thumbs-up and thumbs-down icon. If participants give a thumbs-down to a photo of a potential partner, they move on to the next photo. If participants give a thumbs-up to a photo, a starred rating scale appears on the screen, and participants are asked to rate the attractiveness of the potential partner on a 5-star scale before moving to the next photo. After viewing all 60 photos, participants are informed they have been matched with several partners and will get an opportunity to participate in 2 speed dates.

**Question and answer (Q&A) speed date**

Speed dates proceed in a structured and predetermined manner. Speed date partners are selected by the app based on the highest starred ratings that participants provide to the photos viewed in the aforementioned review of potential partners. Before commencing a speed date, participants rate the partner’s physical attractiveness and personality and their interest in meeting their partner in person. Each speed date is then conducted using “G-Date’s patent pending Q&A system,” in which the participant and their partner respond to questions designed to maximize the assessment of a partner’s compatibility. During the Q&A date, a question appears on the screen (eg, “Based solely on your last date, what would that person say about you?”), and participants have 90 seconds to text their response to the question. After time elapses, participants see their response delivered to their partner and receive their partner’s response to the same question, which presumably had been typed at the same time. After the third Q&A, participants can end or continue the speed date, and they are given the opportunity to end or continue the speed date after each subsequent Q&A. In the speed date with the safe partner, none of the partner’s Q&A responses contain risk cues. In the speed date with the risky partner, the first 2 responses to the Q&As do not contain risk cues, but the third and all subsequent responses do; therefore, every participant is exposed to at least 1 risky response from the risky partner and given the opportunity to end the speed date afterward. If participants remain on the risky date, every subsequent response contains a risk cue. After the 12th Q&A, the speed date is automatically terminated. After each speed date, participants rate their partner on the same 3 attractiveness and interest scales as they had before the speed date.

**Partner comparison**

After the completion of both speed dates, participants are presented with side-by-side photos of their speed date partners and asked to rate each of them on a series of dimensions: vibe, interpersonal style, personality, feeling about their sexual identity, respect of sexual boundaries, and alcohol or drug issues.

#### Script for the Q&A Speed Date

Initial stimuli for the Q&A in the speed date embedded within the app were developed based on the existing literature about SV in MSM, as well as feedback from our consultants with expertise in SV among MSM. One set of stimuli was developed for the risky dating partner and contained SV risk cues, while a second set was developed for the safe dating partner and contained no risk cues. A total of 53 potential Q&As were then developed after extensive discussion with the primary research team and consultants to clarify whether the language would be appropriate for young MSM and contains a realistic degree of risk and safety. Responses containing risk cues included descriptions of the loss of control over alcohol or drug use, antisociality (eg, low empathy, narcissism, and aggression), and internalized homophobia, all empirically documented constructs associated with perpetration. For example, in response to the question, “In a sentence or two, what do you like most about being in a relationship?” the risky partner responds as follows: “tbh the best part is having a guy around who knows it’s his job to drain my balls every day.”

With regard to the safe dating partner, we developed a set of stimuli that contained no risk cues. For example, for the question, “What’s one of the best ways for you to get to know a new partner?” the safe partner responds as follows: “It can be a lot of diff things but I think when they share something they are passionate about like a playlist or a favorite movie it can tell you a lot about them.”

Next, using Prolific (Prolific Academic Ltd), a nationally recruited sample of 102 MSM evaluated all proposed Q&As. Participants were told, “Imagine that you are using a speed-dating app to connect men with other men. Please read the following answers provided by a potential partner to questions about themselves, then rate their answers.”

For each Q&A, participants evaluated (1) how realistic it would be that a man provided this answer on a gay speed-dating app (on a scale of 0 [less realistic] to 10 [more realistic]); (2) how likable the person who potentially wrote the answer is (on a scale of –3 [not at all likable] to +3 [extremely likable]); and (3) how likely the person who wrote the answer would be to engage in verbal, physical, and alcohol or drug-facilitated SV (on a scale of –3 [not at all likely] to +3 [extremely likely]). Participants were also asked whether the person who wrote the response would have (1) substance abuse issues (they have an alcohol or drug problem or cannot seem to control their alcohol or drug use; (2) internalized homonegativity (self-hate; someone is uncomfortable being gay); (3) toxic personality (being narcissistic, lacking empathy, or controlling and intimidating); (4) other negative traits; and (5) none of the above.

Responses were included in the final set of Q&As only if the mean realism score was greater than the midpoint of 5. Moreover, for the risky date, all Q&As included ≥1 of the SV types (ie, verbal, physical, and alcohol or drug-facilitated SV) with mean scores in the positive range and the identification of one of the primary perpetration risk constructs (ie, substance use issues, internalized homonegativity, and toxic personality) across participants. For the safe date, all Q&As included only SV types with mean scores in the negative range and no indication of any perpetration risk constructs. The final Q&A responses included in the risky date were rated significantly higher in terms of the likelihood of engaging in all 3 types of SV compared to the Q&A responses included in the safe date. Taken together, these data led to the development of the aforementioned 2 unique dates: risky and safe.

#### Dating Partner Photos

We relied on artificial intelligence (AI) to initially create 139 racial and ethnically diverse headshots of men who would appear to be in their late teens to late twenties to serve as bogus dating partners. One of our primary reasons for choosing AI rather than actual photos stemmed from ethical concerns about using the photos of people whose physical appearance may be judged negatively and associated with the inappropriate behavior of the risky partner in the Q&A date. All photos were evaluated by the same nationally recruited sample of 102 MSM to provide impressions (on a scale of 0 [less] to 10 [more]) of physical attractiveness, interest in dating, and interest in having sex, as well as perceived age, race, ethnicity, community (eg, bear or muscle guy next door), and sexual position (eg, top, bottom, or versatile). It is important to note that due to the large number of photos and Q&As under review, each participant saw only a subset of photos presented in random order.

Visualization of the photo data revealed a broad range of responses across all items. Further, our app development team suggested we limit the potential options for speed date matching to reduce the potentially unnecessary advanced algorithm work. To this end, we decided to split our potential dates into 2 age groups based on perceived age in pilot testing: 18 to 23 years and 24 to 29 years. We also wanted the potential dates to represent a range of attractiveness and to be racially and ethnically diverse to provide a wide range of options for each participant. Therefore, anyone with mean ratings on attractiveness, interest in meeting, and interest in sex <3.67 was removed from consideration. We next removed any photos with participant comments that noted “distortions” that might suggest these were AI-developed images or any photos identified as too similar to another photo (in actuality, all photos have some degree of similarity based on the generative AI algorithms used). We then chose photos of different racial and ethnic groups that were representative of the demographics of the region where the study took place (eg, 65% White) based on the majority ratings by participants that fit typical categories and omitted any photos that demonstrated low scores on attractiveness and interest scales or higher average age group. This led to a final list of 96 photos for use in the app. However, given our interest in maximizing the number of headshots available for participants to choose from (similar to real-world app use), we included 60 photos for each of the age categories. To this end, of the 96 photos, any photo with a mean age of ≤27.29 years was included in the 18- to 23-year category (n=60, 62.5%), and any photo with a mean age of ≥25.82 was included in the 24- to 29-year category (n=60, 62.5%). This decision led to 12 photos that overlapped in both categories. In consultation with our app development team, we also noted the need to include a group of photos that would represent a “both” category for those participants who noted no preference for age group. Therefore, we included the same 12 overlapping photos and a random selection of 24 of the 60 photos from each of the age categories to form a “no preference” category (n=60, 62.5%). Taken together, this led to 3 groups of 60 photos, and all participants with a preference for partners aged 18 to 23 years, 24 to 29 years, or either saw the same 60 photos, respectively.

To refine the stimulus materials and app, we obtained qualitative feedback from MSM. We sought participants aged 18 to 30 years to best target a high-risk group in terms of exposure to SV. Potential participants were screened using a web-based survey for eligibility before engagement in the study based on the following criteria: (1) being aged between 18 and 30 years, (2) gender identification as a man, (3) sexual contact with a man in the previous 12 months, and (4) having used a DSN app in the last 12 months. The web-based focus groups and interviews lasted approximately 1.5 to 2 hours, and participants were paid US $40 for their time. We conducted 3 focus groups and 2 interviews for a total of 14 participants using an iterative process wherein ongoing data analysis and app development informed subsequent interviews. All discussions were audio recorded and transcribed by the research team and uploaded into Dedoose (Sociocultural Research Consultants LLC) for analysis. Participants had a mean age of 23.5 (SD 1.5) years. Half of the participants self-identified as Black (7/14, 50%), while the others self-identified as White (2/14, 14%) or chose not to report their race or ethnicity (5/14, 36%).

Initial participants were asked to provide perceptions of what constitutes cues in an app that a person or an environment is of high risk (signs that indicate a person will become sexually violent or an environment poses danger) and of low risk (signs that indicate a person or an environment has limited risk). Subsequent, participants were presented with the prototype version of G-Date and asked to provide feedback on (1) the app’s realism; (2) the quality of the stimulus materials, including the bogus candidate profile photos and Q&As; and (3) the feasibility of the cover story. In addition to guided discussion, a self-administered questionnaire was used to gather feedback. Throughout this iterative process, there were ongoing modifications of G-Date by the app developer team to incorporate further feedback about the stimulus materials and app usability.

The second (and ongoing) part of this study examines the content, concurrent, and predictive validities of the G-Date paradigm. Given minority stress and the syndemic situation of MSM, we are assessing the following SV risk factors and their relationship to participant behavior in G-Date: (1) historical exposure to SV, (2) alcohol and drug use or abuse, (3) dating and sexual behaviors, (4) psychological symptoms, and (5) minority stress indicators. G-Date also affords the opportunity to examine 2 related but distinct indices of risk perception: behavioral response (the number of responses tolerated from the bogus dating partner) and threat appraisal or identification (changes in the perception of the bogus dating partner). For content validity, participants’ level of risk perception will be assessed relative to their interaction with the risky versus safe partners. For concurrent validity, participants’ level of risk perception during their interaction with the risky partner will be assessed in association with their endorsement of the aforementioned risk factors for SV. For predictive validity, participants’ level of risk perception during their interaction with the risky partner will be assessed in association with their exposure to SV at the 9-month follow-up.

### Participant Recruitment and Eligibility

Potential participants are being recruited via advertisements on social media platforms and at in-person locations (eg, lesbian, gay, bisexual, and transgender [LGBT] centers and college campuses). Participant eligibility criteria include the following: (1) being aged 18 to 30 years, (2) gender identification as a man, (3) sexual contact with a man in the previous 12 months, and (4) DSN app use in the last 12 months. Any individual who participated in aim 1 is excluded, given their knowledge that G-Date is a simulation. The completion of the G-Date protocol takes approximately 1.5 hours, and participants are paid US $30 for their time.

### Procedure

Data collection occurs within physical laboratory spaces using iPads or over a video-call platform using participants’ iPhones, iPads, or Apple laptops. Participants are told that the experimenter is “part of a team of consultants contracted to beta test a prototype of a new speed-dating app called G-Date, for men who have sex with men*.*” Experimenters explain that they seek to evaluate “the best speed date questions” and want participants’ feedback from their dates that are limited to answering a set of predetermined questions. Participants are provided with a username and password so that they can log into G-Date and engage the paradigm. This entails creating a profile and reviewing photos of potential dating partners (as described in Textbox 1).

After being exposed to photos of 60 potential dating partners, participants are told that based on their preferences and profiles, they can have 2 dates, due to the “limited number of users available in the beta-testing phase.” Participants then begin the first of the 2 dates. Each participant has 1 risky dating partner (risky condition) and 1 safe dating partner (safe condition), as described in the Script for the Q&A Speed Date section. The order of the conditions is randomized. After the G-Date paradigm, participants are asked to complete a battery of questionnaires on Qualtrics (Qualtrics International Inc), which includes a demographic form, and a comprehensive battery of personality and behavioral measures targeting syndemic and minority stress-related factors that have been theoretically or empirically linked with SV (Multimedia Appendix 1). Upon the completion of the questionnaires, participants are debriefed, compensated, and thanked for their participation. To evaluate the predictive validity of the paradigm, participants will be contacted 9 months after their initial visit to the laboratory and asked to retake the battery of questionnaires they originally completed with reference to the time period since their initial study completion.

### Data Analytic Plan for Aim 2

Before testing our hypotheses, adherence to distributional assumptions will be examined. When the assumptions of linear models are untenable, we will use transformations, nonparametric procedures, or generalized linear models. The family of generalized linear models will be determined based on the distribution of the data, although it will likely be either a Poisson or a negative binomial model. Missing data will be handled via multiple imputation [87], and results will be reported after considering both findings obtained through listwise deletion and multiple imputation. To evaluate the validity of G-Date, we have 3 hypotheses that map onto 3 different types of statistical models.

Our first set of hypotheses addresses the content validity and premise of the G-Date paradigm, that is, that participants will perceive and respond to the risky date differently than the safe date. Hypothesis 1 is that participants will engage in fewer Q&As with the risky date than with the safe date. Hypothesis 2 is that there will be larger pre to post evaluation declines in attractiveness and desirability for the risky date than for the safe date. To evaluate these hypotheses, we will conduct paired 1-tailed *t* tests. If the normality assumption is untenable, we will use the Wilcoxon signed rank test or transform the data.

Our second set of hypotheses aims to address the concurrent validity of the G-Date paradigm by evaluating whether participants who engage longer in the Q&A date and more positively evaluate the risky date are rated higher in the various syndemic and minority stress-related characteristics associated with exposure to SV. Hypothesis 3 is that the number of Q&As participants engage in with the risky date will correlate with and be predicted by participants’ historical exposure to SV, alcohol and drug use or abuse, risky sexual behaviors, psychological symptoms, and minority stress. Hypothesis 4 is that the extent of the pre- to postdate questionnaire declines will correlate with and be predicted by participants’ historical exposure to SV, alcohol and drug use or abuse, risky sexual behaviors, psychological symptoms, and minority stress. For some of these variables (eg, history of exposure to SV), we expect many individuals to endorse a lack of experience. Therefore, standard linear models (which assume normality) will not be appropriate. Rather, a Poisson generalized linear model, a negative binomial model, or a zero-inflated model will better match the distribution of the data. To determine which distribution family is appropriate, we will conduct likelihood ratio tests, supplemented with Akaike information criterion and Bayesian information criterion comparisons, as well as residual analyses.

The third set of hypotheses will evaluate the predictive validity of the G-Date paradigm. Participants who engage longer in the Q&As and more positively evaluate the risky date should be at a higher risk for future SV exposure while accounting for historical SV exposure. Hypothesis 5 is that the number of Q&As participants engage in with the risky date will correlate with and predict 9-month exposure to SV. Hypothesis 6 is that the extent of the pre- to postdate questionnaire declines will correlate with and predict 9-month exposure to SV. As in the previous hypotheses, many of these variables are expected to follow a nonstandard (ie, nonnormal) distribution. The results of hypotheses 3 and 4 will inform the selection of both the type of model for hypotheses 5 and 6 and priors for the parameters in a Bayesian analysis.

Given that the targeted sample size (n=325) is only slightly more than the estimated sample size required to detect statistical significance via a priori power analyses (n=275), a Bayesian analysis will increase the chances of the sample size being adequate. The prior distribution for the Bayesian analysis will be estimated from the relationship between history of exposure to SV and risk perception. Provided the relationship between history of exposure to SV and risk perception yields similar parameters to the relationship between 9-month exposure to SV and risk perception, the Bayesian results will yield more precise estimates than traditional (likelihood or frequentist-based) procedures. However, given the unfamiliarity most users have with Bayesian analysis, we will perform the analysis in three ways: (1) using traditional (likelihood or frequentist-based) statistical procedures; (2) using Bayesian analysis with priors obtained from the relationship between past exposure to SV and risk perception; and (3) using a Bayesian analysis using “uninformative” priors, which are prior estimates of the parameter that will heavily weight the data in estimating the parameters, rather than the user-specified priors. The results from these 3 models will serve as a sensitivity analysis; they will be presented side-by-side in an effort to maximize transparency and highlight differences (if any) in the scientific conclusions reached as a function of the models’ assumptions. In addition, it is expected that some individuals will drop out before the completion of the study. As mentioned previously, all the available data will be used to generate a multiple imputation model to impute the missing values. The results from each imputation will be averaged using imputation rules proposed by Rubin [87], and these aggregated results will be presented alongside the more common method of listwise deletion.

## Results

Funding for this project began on September 10, 2020. Development of the G-Date app commenced in October 2020. Enrollment of participants for aim 2 commenced in December 2022. As of May 2024, a total of 245 participants have completed the protocol. Participant recruitment is expected to continue through August 2024, and data collection is expected to be completed in May 2025 to allow for a 9-month follow-up. Final results of the project are expected to be submitted for publication in December 2025.

## Discussion

### Principal Findings

Findings from the study will test G-Date’s content, concurrent, and predictive validities. With respect to content validity, we anticipate that participants will rate the risky partner more negatively and terminate the Q&A date with the risky partner sooner than that with the safe partner. With respect to concurrent validity, we anticipate that syndemic and minority stress-related factors such as prior exposure to SV and substance use will impact participants’ ratings and termination of the risky versus safe dates such that greater syndemic and minority stress will be associated with impairments in risk perception. Finally, with respect to predictive validity, we anticipate that participants’ risk perception during G-Date will predict exposure to SV at the 9-month follow-up.

### Comparison With Prior Work

Our prior work in SV risk perception used a web-based speed-dating paradigm (EduDate) designed for a desktop or laptop computer reflecting the technology of the era in which it was developed. G-Date represents an advance in that it is designed as a mobile app and mimics contemporary DSN apps used by MSM. Further, the stimuli of the EduDate paradigm were developed for use with heterosexual women, while the content of G-Date was developed specifically for use with MSM, an understudied but high-risk population for exposure to SV.

### Strengths

This proposal has several methodological strengths. G-Date is built on a software platform similar to that of popular dating apps; therefore, its appearance and general functionality from setting up a profile to swiping and rating photos of potential partners can be easily understood and used by participants with even casual prior experience with DSN apps. The risk cues embedded into the risky date are derived from the empirical literature, while the language and context in which the cues are presented in the Q&A date were vetted and shaped by discussions with consultants and focus groups and found in pilot testing to be realistic and indicative of risky content. The use of AI to generate photos allowed for a diverse range of attractive headshots of men from varied ethnic backgrounds without the ethical concerns of using actual photos, in which case, the appearance of the men in the photos would be judged and the simulated behavior of the men in the Q&A date could be expected to be seen negatively. Finally, we embedded manipulation and suspiciousness checks that will allow for the flagging of data from participants who did not attend to the risk cues or who did not believe the cover story for the protocol.

### Limitations

There are a number of methodological and practical challenges involved in the undertaking of this project. First, a methodological issue common to laboratory analogs is external validity. Participants may behave differently on G-Date from how they might ordinarily behave when using a DSN app in the community. While G-Date stimuli were pilot-tested for validity, there may be different contextual factors related to participation in the study (eg, beta testing an app for compensation and the presence of an experimenter) that influence participants to behave with heightened or reduced caution. A second related external validity concern is the generalizability of the participants’ risky behavior in G-Date to risky decision-making during real-world in-person dates or hookups. It is possible that participants who engage in more risk in G-Date are, in fact, more cautious when it comes to meeting a new web-based acquaintance in person. We hope to be able to address these potential limitations through the 9-month follow-up with participants, when we will assess their subsequent DSN behavior, exposure to SV, and substance abuse, among other risk variables. A third potential limitation could be a lack of SV experiences reported by participants both initially and at the 9-month follow-up. This would reduce our ability to explore G-Date’s predictive and concurrent validities. A possible remedy for this problem would be to increase the number of participants and identify a more “at-risk” sample, such as specific subgroups of MSM. A related predictive validity concern is extensive attrition at the 9-month follow-up. To this end, we plan to periodically remind participants about the 9-month follow-up and to provide a monetary incentive at the 9-month follow-up.

### Future Directions

Upon the successful validation of G-Date, we anticipate using the refined paradigm on an ongoing basis to elucidate the range and relative strength of risk factors that can inform SV prevention endeavors, especially the study of risk detection under the effects of alcohol intoxication. For example, we anticipate embedding G-Date into an alcohol administration protocol to parse the unique influence of intoxication, as well as the nexus of intoxication and other relevant alcohol or drug use factors. We also believe G-Date can be used within prevention programs. First, because G-Date will serve as a validated measure of risk perception, it can be used as a tool to measure the success of relevant intervention programs. For example, G-Date behaviors could be evaluated both before and after the intervention, and a successful program would demonstrate an increase in participant risk perception over time. Second, G-Date might be used as a stand-alone mechanism for prevention programming. For example, the paradigm could be used to educate participants about navigating sexual risk and communication in DSN app environments. Therefore, participants engaging in G-Date could be reinforced for decisions that could protect them from SV and educated about decisions that could increase the risk for SV in real time.

### Conclusions

This project aims to (1) design a new laboratory paradigm in the form of a DSN app called G-Date that can be used to investigate SV risk perception in MSM and (2) evaluate G-Date’s content, concurrent, and predictive validities by examining the relationship between participant engagement in the app with risky and safe dating partners and their status on a battery of syndemic and minority stress-related factors. The findings have the potential to provide a new methodological tool for basic and applied researchers and advance the understanding of crucial factors related to SV risk perception in MSM.

## Appendix

Multimedia Appendix 1 Summary of measures administered to participants.

Multimedia Appendix 2 Peer-review reports from the Health Disparities and Equity Promotion (HDEP) Study Section - Healthcare Delivery and Methodologies Integrated Review Group - Center for Scientific Review (National Institutes of Health, USA).

## Abbreviations

AI: artificial intelligence
DSN: dating and sexual networking
IPV: intimate partner violence
LGBT: lesbian, gay, bisexual, and transgender
MSM: men who have sex with men
Q&A: question and answer
SV: sexual violence

## Data Availability

The data sets generated during this study will be available in the NIMH Data Archive (NDA) repository [88].
