# Supplementary material for: Assessment of Sexual Violence Risk Perception in Men Who Have Sex With Men: Proposal for the Development and Validation of “G-Date”
Source: JMIR Res Protoc. 2024 Aug 19;13:e57600. doi: 10.2196/57600 (PMC11369526; doi:10.2196/57600)
Supplement: Multimedia Appendix 1 [file resprot_v13i1e57600_app1.docx]

**Multimedia Appendix**

| **Table 1:** Summary of measures Administered to participants (organized by category) | |
| --- | --- |
| **Category** | **Measures** |
| Victimization History | Childhood Trauma Questionnaire-Short Form (CTQ-SF)[67] |
|  | Childhood Sexual Abuse Questionnaire [68] |
|  | Sexual Experiences Survey Short Form Victimization (SES-SFV)[69] |
|  | Intimate Partner Violence among Gay and Bisexual Men Scale (IPV-GBM)[70] |
| Alcohol/Drug Use | Alcohol Use Disorders Identification Test (AUDIT)[71] |
|  | Drug Use Disorders Identification Test (DUDIT)[72] |
|  | NIAAA Recommended Alcohol Questions[73] |
| Dating and Sexual Behaviors | Sexual Risk Survey (SRS)[74] |
|  | Race-Based Sexual Preferences and Stereotypes (RBSPS)[75] |
|  | Sexual Self-Esteem[76] |
|  | Approach/Avoidance Motivations for Sex[77] |
|  | Online Dating Experiences and Perceptions[78] |
|  | Attitudes Toward Looking for Partners via Dating Apps Scale[79] |
|  | Intent to Use Dating Apps Scale[79] |
| Psychological Symptoms | Overall Anxiety Severity and Impairment Scale (OASIS)[80] |
|  | Overall Depression Severity and Impairment Scale (ODISIS)[81] |
|  | PTSD Checklist-DSM 5 (PCL-5)[82] |
| Minority Stress | Internalized Homophobia Scale Short Form (IHP-5)[83] |
|  | Modern Racism Scale (MRS)[84] |
|  | Everyday Discrimination Scale[85] |
|  | Internalized Homophobia Scale (IHP)[86] |
|  | LGBT Community Affiliation Scale[87] |
|  | Loneliness Scale (ULS-8)[88] |
| Control Measures | Dating Self-Protection Against Rape Scale (DSPARS)[89] |
|  | Race-Based Sexual Preferences and Stereotypes (RBSPS)[75] |

**Figure 1.** Study aims and procedures

Aim 2: Investigate DSN App Validity

Aim 1: Develop and Refine DSN App, Protocol, & Stimulus Materials

Production of G-Date App & Protocol

Creation of G-Date Stimulus Materials

Experimental Protocol Administration & App-Based Data Collection

Recruitment of Participants, Eligibility Screening & Informed Consent

Refinement of App, Protocol, & Materials: Finalization of G-Date for Aim 2

G-Date Protocol Administration

9-Month Follow-Up

**
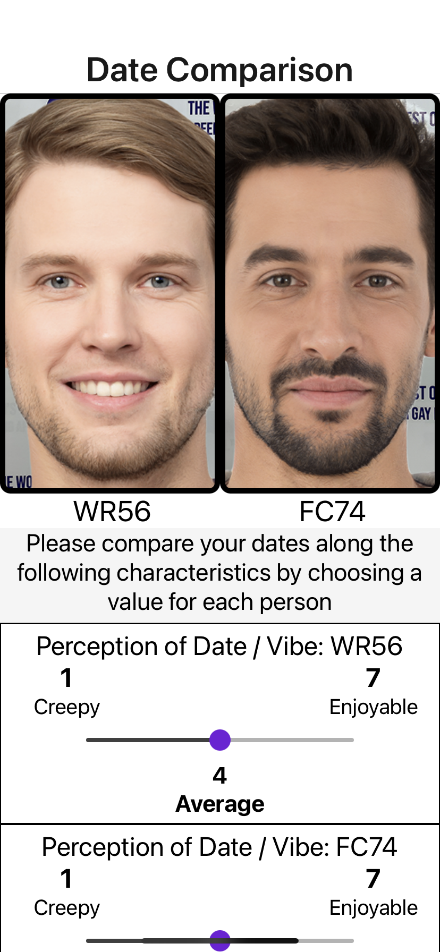

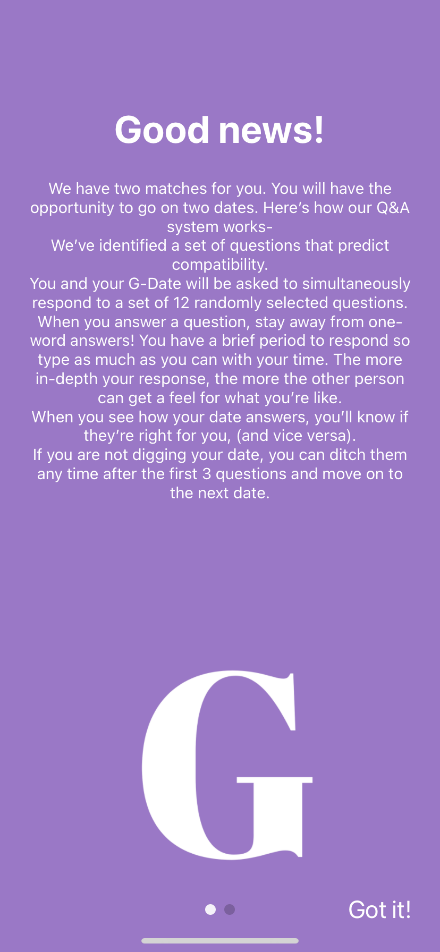

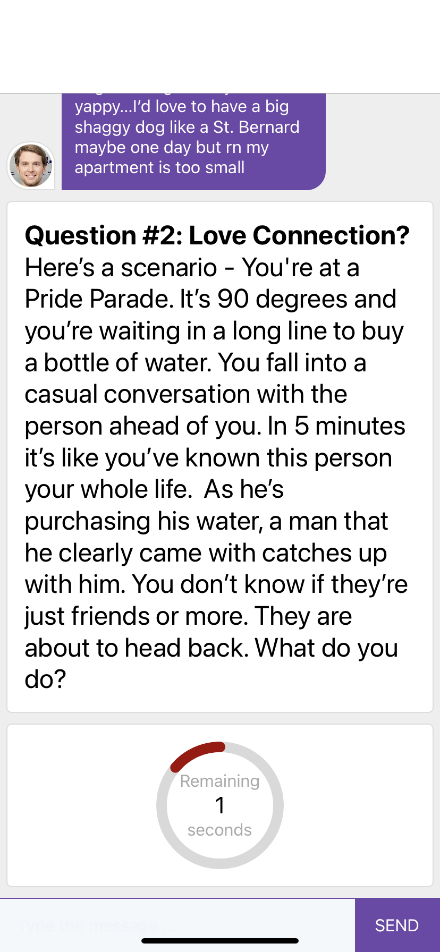
Figure 2.** Screenshots of protype G-Date app
