# Supplementary material for: Assessment of Sexual Violence Risk Perception in Men Who Have Sex With Men: Proposal for the Development and Validation of “G-Date”
Source: JMIR Res Protoc. 2024 Aug 19;13:e57600. doi: 10.2196/57600 (PMC11369526; doi:10.2196/57600)
Supplement: Multimedia Appendix 2 [file resprot_v13i1e57600_app2.pdf]

SUMMARY STATEMENT
(Privileged Communication)

PROGRAM CONTACT:
Dr. Robert Freeman
301443-8820
rfreeman@mail.nih.gov

Release Date: 03/27/2020
Revised Date:

Application Number: 1 R15 AA028637-01

Principal Investigator

ANGELONE, DAVID JASON

Applicant Organization: ROWAN UNIVERSITY

Review Group: HDEP
Health Disparities and Equity Promotion Study Section

Meeting Date: 02/26/2020
Council: MAY 2020
Requested Start: 07/01/2020
RFA/PA: PAR18-714
PCC: AE FD

Project Title: The development and validation of a novel paradigm for assessing sexual assault risk perception in MSM
SRG Action: Impact Score:21
Next Steps: Visit https://grants.nih.gov/grants/next_steps.htm
Human Subjects: 30-Human subjects involved - Certified, no SRG concerns
Animal Subjects: 10-No live vertebrate animals involved for competing appl.
Gender: 3U-Only men, scientifically unacceptable
Minority: 1A-Minorities and non-minorities, scientifically acceptable
Age: 7A-Only Adults, scientifically acceptable

Project Year	Direct Costs Requested	Estimated Total Cost
1	299,998	458,153
TOTAL	299,998	458,153

ADMINISTRATIVE BUDGET NOTE: The budget shown is the requested budget and has not been adjusted to reflect any recommendations made by reviewers. If an award is planned, the costs will be calculated by Institute grants management staff based on the recommendations outlined below in the COMMITTEE BUDGET RECOMMENDATIONS section.

1R15AA028637-01 Angelone, David

INCLUSION OF WOMEN PLAN UNACCEPTABLE

RESUME AND SUMMARY OF DISCUSSION: The application proposes to tailor and validate an existing laboratory analogue of sexual violence risk perception among men who have sex with men (MSM). This highly significant study addresses the important public health issue of sexual violence associated with alcohol and drug use among an understudied population. Reviewers found the development of an app based on an existing model as innovative. The examination of prior research is rigorous and informs the well-described concurrent and predictive validity studies in the approach. The inclusion of students is appropriately integrated into the overall approach. Minor weaknesses noted by reviewers include the limited justification to exclude measures of implicit bias given the racial and ethnic diversity of the sample. Some reviewers questioned the exclusion of transmen or those transitioning based on their risk of sexual violence. Although the use of qualitative methods to inform the development of the app is a strength, the selection of focus groups as a method is not clearly articulated. Overall, reviewers were enthusiastic about the potential of the application for high scientific impact in the field.

DESCRIPTION (provided by applicant): The development and validation of a novel paradigm for assessing sexual violence risk perception in MSM. Sexual violence (SV) is a significant problem for sexual minorities, including men who have sex with men (MSM). The limited research in this area suggests SV is associated with a host of syndetic conditions such as alcohol and drug use, mental health issues, and sexual risk behaviors. These factors tend to cluster and interact to worsen each of the other conditions. Unfortunately, while much work has been conducted to examine these factors in heterosexual women, there is lack of research examining MSM, especially their ability to perceive the potential for SV risk. Further, MSM are active users of dating and sexual network (DSN) applications and this technology has demonstrated usefulness for creating safe spaces for MSM to meet and engage sexual and romantic partners. However, there is mounting data demonstrating that DSN app use is associated with increased risk for SV, especially given the higher likelihood of using alcohol and other drugs before sex. On the other hand, some researchers have demonstrated that DSN technology can be harnessed as a prevention tool for HIV; unfortunately, no such work has progressed with regard to SV. As such, the current project will use qualitative and quantitative methods to tailor an existing laboratory analogue of SV risk perception in women to be used with MSM. This paradigm will be subjected to a rigorous validation study to confirm its usefulness in predicting risk for SV, with the potential for use in future prevention endeavors, including alcohol administration protocols. During the first year of the project, a computer scientist will create the initial DSN app ("G-Date") and incorporate ongoing feedback about the usability, feasibility, and realism of this research tool from a representative sample of MSM. The initial risk cues embedded in the paradigm will include individual and relationship-level risk factors for SV perpetration that include: Alcohol/drug use, masculinity-related factors, and minority stress, including internalized homophobia. During the second and third years of the project, MSM will engage an experimental study to determine the content, concurrent, and predictive validity of this novel laboratory paradigm. In short, we will evaluate whether certain correlates of SV (e.g., history of SV, alcohol and drug use) affect the ability of MSM to detect risk for SV within the G-Date paradigm and in the "real world." It is hoped that G-Date will not only assist the understanding of factors associated with SV risk, but also serve as a useful step in creating tools to enhance prevention efforts in this susceptible population. Given the goals of the R15 mechanism, student RAs will play an active role in all participant recruitment, focus group development and delivery, paradigm development, and experimental evaluation, as well as presentation and publication of the findings. RAs will receive mentoring from experts in SV, alcohol, sexual minority health, software engineering, and qualitative/quantitative research methods, which will prepare them for careers in in their respective fields.

PUBLIC HEALTH RELEVANCE: Sexual violence (SV) is highly prevalent for men who have sex with men (MSM), and this risk is associated with serious negative health outcomes. The proposed project will use qualitative and quantitative methods to tailor an existing laboratory analogue of SV in heterosexual women to be used with MSM. Subsequently, this paradigm will be subjected to a rigorous validation study to confirm its usefulness in predicting risk for SV, with the potential for use in future prevention endeavors.

CRITIQUE 1

Significance: 3
Investigator(s): 3
Innovation: 4
Approach: 4
Environment: 1

Overall Impact: This R15 proposal seeks to develop a laboratory protocol for education and intervention for sexual violence among MSM. The study team is well qualified and presents a sound rationale for the value of the study. The study design is quite good and builds for a similar approach for SV among cis-gender heterosexual women. The plan to assess feasibility and validate the new app that will be developed is sound. There were moderate weaknesses in some areas. The investigators failed to account for implicit bias in their design, and to present a sound rationale for the exclusion of transmen who have sex with men. Despite these deficits, the overall proposal is strong and includes a good for the inclusion of students.

1. Significance
Strengths
- Sexual violence (SV) is a major public health issue.
- MSM SV is a major issue that we lack true understanding of and have few tools to intervene.
- Syndemic factors may impact MSM ability to see risks of SV.
- Apps and digital technologies are a growing area of SV risk.

Weaknesses
- While the investigators discuss MSM SV among ethnic/racial minorities, the intersectional nature of MSM SV seems to be underappreciated.

2. Investigator(s)
Strengths
- PI Angelone is a psychologist with prior expertise in sexual violence research. He has prior seed and state funding and has served as a consultant on an R44.
- Dr. Fife is a psychologist who will serve as study statistician. Dr. Fife has served in this role on other NIH studies.
- Dr. Mitchell is a psychologist with expertise in SV and has collaborated with Dr. Angelone before. Dr. Mitchell has had prior state and federal service and evaluation grants.
- Dr. Wells is an expert in sexual violence. Dr. Well has served as a Co-I on an NIH R01 and PI for an R03.
- Dr. Kurkovsky brings expertise in mobile app development as a software engineer.

Weaknesses
- The team, despite significant content expertise, does not mention or show experience in mentorship of undergraduates. This is an important component to the R15 mechanism.

3. Innovation

Strengths

- The G-Date app will be built on the model created by the EduDate model which has been used in prior research on SV among heterosexual women. This line of work lends innovation to the proposed study as it indexes a successful prior deployment of a related model.
- The creation of a laboratory paradigm has the potential to create a safe way to educate MSM about SV risk and perception.
- The training afford to undergraduates has the potential to be both scientifically salient and socially engaged – there is a high likelihood that this work will draw students into clinical-research careers.

Weaknesses

- The research paradigm could be better expanded to capture more about intersectionality. Age, masculinity and race/ethnicity among the presented date models on G-Date could be components of risk perception – measures of implicit bias would be helpful in understanding the experience SV risk perception.

4. Approach

Strengths

- The proposal indicates that the research will begin with app development to mirror the EduDate paradigm with translation to the mobile environment and with adaptations for MSM.
- Participant recruitment will take place locally near the university, in Camden and Philadelphia. The investigators propose to use LGBT centers, coffee shops and social media.
- Ages 18-30 will be included who are cis-gender males. Focus groups will be done to guide development of G-Date.
- App feasibility will be established in Phase 2 where 194 cis-gender MSM will be recruited to use the mock app.
- Well established scales will be used to examine the various psychological constructs that are relevant to the study.
- 12-month follow-up will be done.

Weaknesses

- Transmen are excluded from the study. It is unclear why transmen who have sex with men would be excluded given the high rates of SV against this subpopulation – a true intersectional framework would allow for the inclusion (and possible oversampling for analysis) of this population.
- The lack of inclusion of a measure of implicit bias among the participants is a major flaw in this study. Participants are apt to perceive risk in highly racialized terms, and capturing implicit bias is essential to the analysis behind this study.

5. Environment

Strengths

- Rowan University appears to be well positioned to support this study.
- The current resources for Dr. Angelone show a commitment to high quality research.
- The student population is well described and fits well with R15 objectives.

Weaknesses

- None

Study Timeline

Strengths

- None noted by reviewer.

Weaknesses
- None noted by reviewer.

Protections for Human Subjects
Acceptable Risks and/or Adequate Protections
- The investigators have good plans in place for human subject protections.

Data and Safety Monitoring Plan (Applicable for Clinical Trials Only):
Not Applicable (No Clinical Trials)

Inclusion Plans
- Sex/Gender: Distribution not justified scientifically
- Race/Ethnicity: Distribution justified scientifically
- For NIH-Defined Phase III trials, Plans for valid design and analysis: Not applicable
- Inclusion/Exclusion Based on Age: Distribution justified scientifically
- The exclusion of trans-males is not justified scientifically.

Vertebrate Animals
Not Applicable (No Vertebrate Animals)

Biohazards
Not Applicable (No Biohazards)

Applications from Foreign Organizations
Not Applicable (No Foreign Organizations)

Select Agents
Not Applicable (No Select Agents)

Resource Sharing Plans
Not Applicable (No Relevant Resources)

Authentication of Key Biological and/or Chemical Resources
Not Applicable (No Relevant Resources)

Budget and Period of Support
Recommend as Requested

CRITIQUE 2

Significance: 1
Investigator(s): 1
Innovation: 1
Approach: 2
Environment: 1

Overall Impact: This innovative application, has a very strong team, highly relevant pilot data, and proposes a rigorous method for evaluating a highly innovative 21st century paradigm for assessing a very significant problem in MSM (alcohol related dating violence). I am highly enthusiastic about this application. My only minor concern is feasibility of a 12 month follow up in a three-year grant. This is an outstanding study that can and should be done.

1. Significance
Strengths
- Alcohol related dating victimization is a significant public health problem for MSM.
- Tools for assessing this risk are outdated (e.g., laboratory bars).
- Development of an app that assesses this risk is innovative.
- The pilot data presented indicates that they have an app that can be adapted for MSM.
- The premise for this study is strong

Weaknesses
- None

2. Investigator(s)
Strengths
- The PI has a strong background in this area
- The PI has developed the app that will be developed
- The team includes consultants who are leaders in this field

Weaknesses
- None

3. Innovation
Strengths
- This is a very innovative and timely approach to measure risk of dating violence in this population.
- The intensive development to assure that the tool is applicable to different races and ethnicities is important.

Weaknesses
- None.

4. Approach
Strengths
- The approach is highly rigorous and well described.
- The app modification/development approach is well described.
- The outcome assessment will include sufficient data for a larger trial, including data for up to 12 months on dating violence.

Weaknesses
- The 12-month follow up is a strength but in a three-year grant, it may not be feasible.

5. Environment
Strengths
- The team has developed the resources needed to carry out the project.

Weaknesses
- None

Study Timeline
Strengths
- Appropriate

Weaknesses
- Might be challenging to get 12 month follow up in 3 years

Protections for Human Subjects

Acceptable Risks and/or Adequate Protections

- Acceptable

Data and Safety Monitoring Plan (Applicable for Clinical Trials Only):

Acceptable

- Acceptable

Inclusion Plans

- Sex/Gender: Distribution justified scientifically
- Race/Ethnicity: Distribution justified scientifically
- For NIH-Defined Phase III trials, Plans for valid design and analysis: Not applicable
- Inclusion/Exclusion Based on Age: Distribution justified scientifically
- All justified.

Vertebrate Animals
Not Applicable (No Vertebrate Animals)

Biohazards
Not Applicable (No Biohazards)

Applications from Foreign Organizations
Not Applicable (No Foreign Organizations)

Select Agents
Not Applicable (No Select Agents)

Resource Sharing Plans
Acceptable

Authentication of Key Biological and/or Chemical Resources
Not Applicable (No Relevant Resources)

Budget and Period of Support
Recommend as Requested

CRITIQUE 3

Significance: 2
Investigator(s): 1
Innovation: 2
Approach: 3
Environment: 2

Overall Impact: This R15 application is from Rowan University which was recently designated a research institute by New Jersey. The application addresses a significant area of research – sexual violence against men who have sex with men (MSM). The focus of the application is on the development and testing of a laboratory paradigm app to investigate sexual violence risk perception in MSM. The app is based on a similar product developed for heterosexual women (EduDate) and will be modified with a well-developed adaptation plan. The strong research team and the supportive environment at Rowan University lends support to the ability to accomplish the study aims. The PI

(Angelone) has expertise in the area of sexual violence and has worked extensively with undergraduate and graduate students. Additional investigators and consultants provide content and research management experience. The application is innovative in its target population focus and in the extent to which students will be involved. Weaknesses in the approach include the lack of adequate justification for several methods components including the 12-month post-test interval, the extensive pre-post tools to be completed by Aim 2 participants, This project has the potential to impact sexual violence against MSM and the team has the ability to conduct the study.

1. Significance
Strengths
- Sexual violence among MSM is a critical problem.
- Dating and sexual networking (DSN) apps were found to increase identification of risky behaviors for heterosexual women.
- The development of an app for heterosexual women measures threat appraisal via response latency (length of time to determine risk perception) – this will be adapted for the G-Date to be developed for MSM.
- The scientific premise is based on syndetic, response latency, and minority stress constructs.

Weaknesses
- The application did not adequately address if the project would strengthen the research environment of the institution.
- The scientific premise for the 12-month follow-up is not well supported. Preliminary data reported for the EduDate app did not address 12-month predictive validity testing nor is there preliminary data related to the extensive number of data collection tools proposed in Aim 2.

2. Investigator(s)
Strengths
- The PI (Angelone) and Co-I are psychologists who have previous collaborations in the area of sexual violence research and in treating victims of sexual violence.
- Other Co-I's include Dr. Well who has conducted sexual assault research and has experience recruiting sexual minorities. Dr. Kurkovsky has expertise in developing mobile apps and software engineering and has worked with students on these efforts. Statistical support is provided by Dr. Fife.
- Consultants provide additional support. Dr. George has experience working with minorities in the areas of alcohol, sexual risk and sexual violence. Dr. Davis has expertise in sexual-decision making. Dr. Pantalone has expertise in sexual victimization drug abuse, and sexual risk behaviors. Sr. Sitron has expertise in MSM and the use of dating apps. These consultants will be available to support and provide guidance to the investigators.
- Dr. Angelone completed a sabbatical to explore the interaction of alcohol use and expectancies with sexual violence. Dr. Angelone has extensive experience working with undergraduate students in his research lab.

Weaknesses
- None noted

3. Innovation
Strengths
- Development of an app to understand factors related to sexual violence in MSM is an innovative approach. The app allows for real time risk perception assessment.
- Students will be involved in all aspects of the development and testing of the app. Including conducting focus groups and analysis of data. An innovative aspect is the context and the opportunity students will have to work with a vulnerable, minority population.

Weaknesses

- Given the extensive number of apps available, developing another app is no longer an innovative approach.

4. Approach

Strengths

- Students will create the G-Date app with supervision from the computer science Co-I (Kurkovsky).
- The development of 2 ‘chats’ one with a ‘risky’ candidate, and one with a ‘safe’ candidate to allow assessment of risk perception and response latency. Data will be collected on number of responses in the risky vs safe conditions, and changes in ratings on desirability and interest in risky dating candidates – allowing for multiple means to assess risk perceptions.
- Student involvement in Aim 1 include a graduate student research coordinate and 4 paid undergraduate research assistants. Students will receive training in the content area as well as in research ethics and methods.
- A strength is the assessment of feasibility, usability, and acceptability of the app with 40 additional participants in Aim 1.
- Aim 2 assessment of content, concurrent, and predictive validity assures the app is well developed and has educational value. The methods are adequately described.
- Aim 2 is adequately powered.

Weaknesses

- Aim 1 participation is limited to focus groups, the rationale for not including interviews as an option for those not able or willing to participate in a group meeting was not described.
- Rationale for 5 focus groups not provided, the application lacks adequate discussion related to data saturation.
- Aim 1 data analysis is under-developed and lacks specifics related to student involvement.
- Neither the rational for, nor the psychometric properties of the extensive number of tools proposed in Aim 2 are discussed.
- Rationale for a 12-month follow-up is not provided, nor are retention methods described.

5. Environment

Strengths

- Rowan University has recently been designated as a research institution by the State of New Jersey.
- The pool of undergraduate students will be drawn from the 16,120 student body. Efforts will be made to include diverse and underrepresented students in the project.
- Dr. Angelone’s lab areas are adequate to conduct all phases of the study.
- Adequate evidence was provided that student’s at Rowan university are likely to pursue careers in health-related sciences

Weaknesses

- The environment at Central Connecticut University where the development of the app will occur was not addressed.

Study Timeline

Strengths

- None noted by reviewer.

Weaknesses

- None noted by reviewer.

Protections for Human Subjects

Acceptable Risks and/or Adequate Protections
- Acceptable risks and adequate protections

Data and Safety Monitoring Plan (Applicable for Clinical Trials Only):
Acceptable

Inclusion Plans
- Sex/Gender: Distribution justified scientifically
- Race/Ethnicity: Distribution justified scientifically
- For NIH-Defined Phase III trials, Plans for valid design and analysis: Not applicable
- Inclusion/Exclusion Based on Age: Distribution justified scientifically

Vertebrate Animals
Not Applicable (No Vertebrate Animals)

Biohazards
Not Applicable (No Biohazards)

Applications from Foreign Organizations
Not Applicable (No Foreign Organizations)

Select Agents
Not Applicable (No Select Agents)

Resource Sharing Plans
Unacceptable
- Not provided.

Authentication of Key Biological and/or Chemical Resources
Not Applicable (No Relevant Resources)

Budget and Period of Support
Recommend as Requested
- Faculty at Rowan University are on 10-month contracts, it is unclear if effort includes summer salary.

THE FOLLOWING SECTIONS WERE PREPARED BY THE SCIENTIFIC REVIEW OFFICER TO SUMMARIZE THE OUTCOME OF DISCUSSIONS OF THE REVIEW COMMITTEE, OR REVIEWERS' WRITTEN CRITIQUES, ON THE FOLLOWING ISSUES:

PROTECTION OF HUMAN SUBJECTS: ACCEPTABLE

INCLUSION OF WOMEN PLAN: UNACCEPTABLE
Reviewers noted the exclusion of transmen or transmen in transition is not scientifically justified. The scientific rationale for the distribution of transmen in the sample should be clearly described.
INCLUSION OF MINORITIES PLAN: ACCEPTABLE

INCLUSION ACROSS THE LIFESPAN: ACCEPTABLE

COMMITTEE BUDGET RECOMMENDATIONS: The budget was recommended as requested.

Footnotes for 1 R15 AA028637-01; PI Name: Angelone, David Jason

NIH has modified its policy regarding the receipt of resubmissions (amended applications).See Guide Notice NOT-OD-18-197 at https://grants.nih.gov/grants/guide/notice-files/NOT-OD-18-197.html. The impact/priority score is calculated after discussion of an application by averaging the overall scores (1-9) given by all voting reviewers on the committee and multiplying by 10. The criterion scores are submitted prior to the meeting by the individual reviewers assigned to an application, and are not discussed specifically at the review meeting or calculated into the overall impact score. Some applications also receive a percentile ranking. For details on the review process, see http://grants.nih.gov/grants/peer_review_process.htm#scoring.

MEETING ROSTER

Health Disparities and Equity Promotion Study Section
Healthcare Delivery and Methodologies Integrated Review Group
CENTER FOR SCIENTIFIC REVIEW
HDEP
02/26/2020 - 02/27/2020

Notice of NIH Policy to All Applicants: Meeting rosters are provided for information purposes only. Applicant investigators and institutional officials must not communicate directly with study section members about an application before or after the review. Failure to observe this policy will create a serious breach of integrity in the peer review process, and may lead to actions outlined in NOT-OD-14-073 at https://grants.nih.gov/grants/guide/notice-files/NOT-OD-14-073.html and NOT-OD-15-106 at https://grants.nih.gov/grants/guide/notice-files/NOT-OD-15-106.html, including removal of the application from immediate review.

CHAIRPERSON(S)

BOUTIN-FOSTER, CARLA, MD
ASSOCIATE DEAN
OFFICE OF DIVERSITY EDUCATION AND RESEARCH
SUNY DOWNSTATE MEDICAL CENTER
BROOKLYN, NY 11203

MEMBERS

ALLEN, HEIDI LYNN, PHD
ASSOCIATE PROFESSOR
SCHOOL OF SOCIAL WORK
COLUMBIA UNIVERSITY
NEW YORK, NY 10027

ARCOLEO, KIMBERLY JOAN, PHD, MPH *
ASSOCIATE PROFESSOR
ASSOCIATE DEAN FOR RESEARCH
SCHOOL OF NURSING
UNIVERSITY OF ROCHESTER
ROCHESTER, NY 14642

ARRIOLA, KIMBERLY, PHD
PROFESSOR
DEPARTMENT OF BEHAVIORAL SCIENCES AND HEALTH EDUCATION
ROLLINS SCHOOL OF PUBLIC HEALTH OF EMORY UNIVERSITY
ATLANTA, GA 30322

BREGA, ANGELA GWEN, PHD *
ASSOCIATE PROFESSOR
CENTERS FOR AMERICAN INDIAN AND ALASKA NATIVE HEALTH
COLORADO SCHOOL OF PUBLIC HEALTH
UNIVERSITY OF COLORADO DENVER
AURORA, CO 80045

BROWN, ARLEEN F, MD, PHD *
PROFESSOR
DEPARTMENT OF MEDICINE
UNIVERSITY OF CALIFORNIA, LOS ANGELES
LOS ANGELES, CA 90024

CABASSA, LEOPOLDO J, PHD
ASSOCIATE PROFESSOR
BROWN SCHOOL OF SOCIAL WORK
WASHINGTON UNIVERSITY IN ST LOUIS
ST. LOUIS, MO 63130

DESAI, JAY R, PHD
RESEARCH INVESTIGATOR
HEALTH PARTNERS INSTITUTE
BLOOMINGTON, MN 55425

GENTILE, DANIELLE, PHD, BS *
HEALTH SERVICES RESEARCHER; ASSISTANT PROFESSOR OF MEDICINE
DEPARTMENT OF SUPPORTIVE ONCOLOGY
LEVINE CANCER INSTITUTE
ATRIUM HEALTH
THE CENTER FOR SUPPORTIVE ONCOLOGY
CHARLOTTE, NC 28204

GIURGESCU, CARMEN, BS, MS, POSTDOCTOR, PHD *
ASSOCIATE PROFESSOR
COLLEGE OF NURSING
THE OHIO STATE UNIVERSITY
COLUMBUS, OH 43210

GOLDBACH, JEREMY THOMAS, AB, MA, PHD *
ASSOCIATE PROFESSOR
UNIVERSITY OF SOUTHERN CALIFORNIA
SCHOOL OF SOCIAL WORK
LOS ANGELES, CA 90089

GONZALEZ-GUARDA, ROSA MARIA, MPH, MSN, PHD
ASSOCIATE PROFESSOR
DOROTHY L POWELL TERM CHAIR OF NURSING
DUKE UNIVERSITY SCHOOL OF NURSING
DUKE UNIVERSITY
DURHAM, NC 27710

GRAVLEE, CLARENCE C, PHD
ASSOCIATE PROFESSOR
DEPARTMENT OF ANTHROPOLOGY
UNIVERSITY OF FLORIDA
GAINESVILLE, FL 32611

JUDD, SUZANNE E, MPH, PHD
PROFESSOR
DEPARTMENT OF BIOSTATISTICS
SCHOOL OF PUBLIC HEALTH
UNIVERSITY OF ALABAMA AT BIRMINGHAM
BIRMINGHAM, AL 35205

KARNIK, NIRANJAN, MD, PHD
VICE CHAIR PSYCHIATRY
DEPARTMENT OF PSYCHIATRY
RUSH UNIVERSITY MEDICAL CENTER
CHICAGO, IL 60612

KILLIAN, MICHAEL, BS, MS, PHD *
RESEARCH SCIENTIST, CENTER FOR THE STUDY AND
PROMOTION OF COMMUNITIES, FAMILIES AND CHILDREN
COLLEGE OF SOCIAL WORK
FLORIDA STATE UNIVERSITY
TALLAHASSEE, FL 32306

MCDONELL, MICHAEL G, PHD
ASSOCIATE PROFESSOR
DEPARTMENT OF MEDICAL EDUCATION
AND CLINICAL SCIENCES
ELSON S FLOYD COLLEGE OF MEDICINE
WASHINGTON STATE UNIVERSITY
SPOKANE, WA 99210

MCDONNELL, KAREN ANN, BA, PHD *
ASSOCIATE PROFESSOR AND VICE CHAIR
DEPARTMENT OF PREVENTION AND COMMUNITY HEALTH
SCHOOL OF PUBLIC HEALTH
GEORGE WASHINGTON UNIVERSITY
WASHINGTON, DC 20037

NORRIS, ANNE E, PHD, MS, BS, BSN, FAAN *
PROFESSOR
SCHOOL OF NURSING AND HEALTH STUDIES
UNIVERSITY OF MIAMI
MIAMI, FL 33146

NUNEZ-SMITH, MARCELLA, MD
ASSOCIATE PROFESSOR OF MEDICINE PUBLIC HEALTH
AND MANAGEMENT
YALE UNIVERSITY
NEW HAVEN, CT 06520

ORNELAS, INDIA JANE, MPH, PHD *
ASSOCIATE PROFESSOR
HEALTH SERVICES
SCHOOL OF PUBLIC HEALTH
UNIVERSITY OF WASHINGTON
SEATTLE, WA 98195

OWUSU, CYNTHIA, BS, MD, MS *
ASSOCIATE PROFESSOR, DEPARTMENT OF MEDICINE,
SCHOOL OF MEDICINE
MEMBER, CANCER PREVENTION CONTROL AND
POPULATION RESEARCH PROGRAM
CASE COMPREHENSIVE CANCER CENTER
CASE WESTERN RESERVE UNIVERSITY
SOLON, OH 44139

POLIVKA, BARBARA J, MSN, PHD, BSN
ASSOCIATE DEAN AND RESEARCH PROFESSOR
SCHOOL OF NURSING
UNIVERSITY OF KANSAS
KANSAS CITY, KS 66160

QUINN, GWENDOLYN P, PHD
ENDOWED PROFESSOR
DEPARTMENT OF OBSTETRICS AND GYNECOLOGY
DEPARTMENT OF POPULATION HEALTH
SCHOOL OF MEDICINE
NEW YORK UNIVERSITY
NEW YORK, NY 10016

RASMUS, STACY M, PHD
DIRECTOR AND RESEARCH ASSOCIATE PROFESSOR
INSTITUTE OF ARCTIC BIOLOGY
CENTER FOR ALASKA NATIVE HEALTH RESEARCH
UNIVERSITY OF ALASKA FAIRBANKS
FAIRBANKS, AK 99775

ROSENZWEIG, MARGARET Q, PHD
PROFESSOR
DEPARTMENT OF ACUTE/TERTIARY CARE
SCHOOL OF NURSING
UNIVERSITY OF PITTSBURGH
PITTSBURGH, PA 15261

SALES, ANNE EVELYN, RN, PHD, MSN, BA *
PROFESSOR
DEPARTMENT OF LEARNING HEALTH SCIENCES
SCHOOL OF MEDICINE
UNIVERSITY OF MICHIGAN
ANN ARBOR, MI 48109

SHALOWITZ, MADELEINE ULLMAN, MD
DIRECTOR OF BIOMEDICAL RESEARCH INFORMATICS
ASSOCIATE PROFESSOR
DEPARTMENT OF PEDIATRICS
UNIVERSITY OF CHICAGO
EVANSTON, IL 60201

SHIPPEE, TETYANA P, PHD *
ASSOCIATE PROFESSOR
DIVISION OF HEALTH POLICY AND MANAGEMENT
SCHOOL OF PUBLIC HEALTH
UNIVERSITY OF MINNESOTA
MINNEAPOLIS, MN 55455

SHUMWAY, MARTHA, PHD
ASSOCIATE PROFESSOR
DEPARTMENT OF PSYCHIATRY
SCHOOL OF MEDICINE
UNIVERSITY OF CALIFORNIA SAN FRANCISCO
SAN FRANCISCO, CA 94143

SIMON, MELISSA A, AB, MPH, MD *
PROFESSOR AND VICE CHAIR
DEPARTMENT OF OBSTETRICS AND GYNECOLOGY
AND PREVENTATIVE MEDICINE
FEINBERG SCHOOL OF MEDICINE
NORTHWESTERN UNIVERSITY
CHICAGO, IL 60611

SINGH, PRAMIL NAND, DRPH, MPH
DIRECTOR AND PROFESSOR
CENTER FOR HEALTH RESEARCH
LOMA LINDA UNIVERSITY
LOMA LINDA, CA 92354

THORPE, ROLAND J JR, PHD
PROFESSOR
DEPARTMENT OF HEALTH, BEHAVIOR, AND SOCIETY
JOHNS HOPKINS BLOOMBERG SCHOOL OF PUBLIC HEALTH
BALTIMORE, MD 21205

WRIGHT, DAVID BRADLEY, BS, MS, PHD *
ASSOCIATE PROFESSOR
DEPARTMENT OF FAMILY MEDICINE
SCHOOL OF MEDICINE
UNIVERSITY OF NORTH CAROLINA AT CHAPEL HILL
CHAPEL HILL, NC 27599

WU, BEI, PHD
DEAN'S CHAIR PROFESSOR
RORY MEYERS COLLEGE OF NURSING
NEW YORK UNIVERSITY
NEW YORK , NY 10010

YEAGER, KATHERINE AYLWARD, BS, BSN, MS, PHD *
NELL HODGSON WOODRUFF SCHOOL OF NURSING
WINSHIP CANCER INSTITUTE OF EMORY UNIVERSITY
EMORY UNIVERSITY
ATLANTA, GA 30322

SCIENTIFIC REVIEW OFFICER

BELLINGER, JESSICA, PHD
SCIENTIFIC REVIEW ADMINISTRATOR
CENTER FOR SCIENTIFIC OF REVIEW
NATIONAL INSTITUTES OF HEALTH
BETHESDA, MD 20892

EXTRAMURAL SUPPORT ASSISTANT

BUTLER, SEAN
LEAD - EXTRAMURAL SUPPORT ASSISTANT
CENTER FOR SCIENTIFIC REVIEW
NATIONAL INSTITUTE OF HEALTH
BETHESDA, MD 20892

* Temporary Member. For grant applications, temporary members may participate in the entire meeting or may review only selected applications as needed.

Consultants are required to absent themselves from the room during the review of any application if their presence would constitute or appear to constitute a conflict of interest.
